# Multiform invasion of life by work among basic education teachers and repercussions on health

**DOI:** 10.11606/s1518-8787.2020054001547

**Published:** 2019-12-23

**Authors:** Jefferson Peixoto da Silva, Frida Marina Fischer

**Affiliations:** I Fundação Jorge Duprat Figueiredo de Segurança e Medicina do Trabalho – Fundacentro. São Paulo, SP, Brasil; II Universidade de São Paulo. Faculdade de Saúde Pública. Departamento de Saúde Ambiental. São Paulo, SP, Brasil

**Keywords:** School Teachers, Health Level, Job Satisfaction, Qualitative Research, Occupational Health

## Abstract

**INTRODUCTION:**

Several studies have pointed to a scenario of precariousness and illness among teachers. However, the way the profession resonates with the personal life of teachers has not received significant attention, even if it is common for them to take work home.

**OBJECTIVE:**

This study investigated the repercussion of work on the everyday life of teachers and its implication on the health-disease process.

**METHODS:**

This is a qualitative study based on individual semi-structured interviews, complemented by a form of sociodemographic characterization. Data were analyzed by thematic coding with the aid of the MAXQDA 12 software. This study included 29 teachers from four public schools of the municipal and state networks of regular and full day education of São Paulo, in addition to the principal of each school.

**RESULTS:**

The results indicated that the illnesses arising from work have been projected on the personal life of teachers. We identified four main forms of manifestation of this type of invasion: continuous link with work by successive frustrations; moral harassment; uninterrupted pending matters; and interference over the private course of life.

**CONCLUSION:**

The social and pathogenic suffering caused by the invasion of life by work pointed to this phenomenon as one of the elements that can help explain the recurrent clinical pictures of illness among teachers.

## INTRODUCTION

Studies analyzing the work-health relationship of teachers in Brazil have shown that the organization and conditions of work of these professionals are hostile, leading them to illness^1^^-^^6^ and/or the abandonment of the profession in its various forms^7^^,^^8^. Among the main forms of illness listed by these studies, we can highlight mental and behavioral disorders; voice and musculoskeletal disorders and cardiovascular disorders. Although these are not the only outcomes, they constitute a picture of typical diseases among teachers, as also observed among teachers from other countries^9^^-^^11^.

The work of teachers is known to have a little visible peculiarity as to the activities conducted outside the school and regular working time, e.g., preparing classes, correcting tests, etc. In general, the world of work has faced significant changes in recent times. Labor demands increasingly advance on private life and people's free time causing negative health impacts^12^. Nevertheless, the literature dedicated to the occupational health of teachers has few discussions on the subject, focusing on the activities conducted at school, that is, a formal workplace.

Our review identified that studies on the quality of life of teachers have grown over the last decade^13^. However, this problem is also not explored in them. On the other hand, the literature on education is focused on the teaching-learning relationship – mediated, above all, by the study of teacher training and educational policies –, and does not seem to have incorporated the health-work relationship into its agenda, whether inside or outside the school.

How would teachers be experiencing and interpreting this reality? What meanings would they be attributing to these experiences? How would work be projecting itself over the everyday personal life of teachers, and to what extent would this be reflecting on clinical pictures of illness of these professionals? The main issue on which this study was based was, therefore, the hypothesis that the work of teachers would be invading their everyday lives in a harmful manner to their health.

## METHODS

This is a qualitative exploratory study performed empirically through semi-structured individual interviews. A form for the sociodemographic characterization of participants was applied for complementary purposes.

The sample consisted of 29 basic education teachers working in four public schools in São Paulo, as well as the four principals of these schools. Two schools were dedicated to regular education (one municipal and one state), and the other two were full day schools (both state). The data were analyzed by thematic coding^14^^,^^15^ using the software MAXQDA, version 12^16^. From the perspective of the theoretical field of occupational health, this study was mainly based on the psychodynamics of work^17^.

This conception understands that the key for comprehending the health-disease process lies on the verge between two types of suffering that affect it: creative and pathogenic. Due to being inherent to the human experience, suffering – as well as pleasure – is ubiquitous, but whether it will lead to illness or will not depend, above all, on the means by which the worker will be able to – or not – creatively transform such suffering. If one is unable to do so, such suffering tends to become pathogenic.

The project was approved by the Research Ethics Committee of the *Faculdade de Saúde Pública* of *Universidade de São Paulo*, under no. 1,553,835, CAAE 54839516.1.0000.5421.

## RESULTS AND DISCUSSION

The mean age of the participants was 45 years, ranging from 29 to 61 years, and the time of experience in teaching ranged between 1 and 37 years (mean was 18 years), constituting a heterogeneous sample, according to the standard of delimited viability. Women composed 55% of the sample.

Regarding the type of employment relationship, 86% were holders of effective position, 10% stable, and 4% had a temporary contract. Regarding marital status, 55% were married and 35% single, whereas 3% were divorced and 7% were under common-law marriage.

Work hours ranged from 11 to 69 weekly hours/classes (mean was 45.6 hours/classes). Among those whose employment relationship allowed them to have other work functions (teachers of full day schools cannot participate in other paid activities during the opening hours of the school), 55% had other jobs and 45% did not. Similar sociodemographic data were observed in other studies^2^^,^^10^^,^^18^^,^^19^.

Regarding the central element of our study (the relationship between work, personal life, and health), 76% of the teachers reported in the characterization form that they took work home. During the interviews, however, this number was much higher, since virtually all interviewees admitted to doing so.

The statements showed the existence of a harmful dynamic of the work of teachers that is manifested from the movement of this profession on their everyday life, something that happens without consent, choice or control on the part of the professional – hence the use of the term invasion –, causing suffering^17^. Several interviewees referred to this phenomenon from the mention of a great difficulty in being able to disconnect from the school and the issues experienced in it, such as the following teacher:

*I take everything to my house […] Everything I talk about is the stress I go through in the classroom. Then my husband says: “Teacher 11A, the school gate has already closed down, so let's talk about other subjects,” but I can't disconnect myself. I should have a little switch that I could turn off, and once I did it, I could forget it, but I can't do it [laughs]. I take it home, and every now and then I talk to my husband, and that's bad because my husband says: “Gee, I already have my job, am I still going to have to take the problems from your work too? From school? Leave them there.” And that's not what happens.* (Teacher 11A)

The invasion of life by work identified in our study, however, does not occur in a single and linear way, but as a complex and multiform phenomenon. Two dimensions of this invasion were found, one material and one non-material.

From a material perspective, the phenomenon took on its best-known form, at least in the case of teachers. This occurred because the most noticeable form of the problem was identified, something considered common in the profession: taking work home. We call it ‘material invasion' due to concerning the material aspect of the profession, e.g., the preparation and correction of tests, assignments, etc.:

*That's the difference, before I went into the education field I was a bank clerk, so when Friday came around I would draw a line: “The weekend is mine, the night is mine.” I cannot do that as a teacher, I do leave part of my material here, but I have a lot of things to do at home. I have to prepare a new test or an assignment, and think about alternatives for the next day, new ways to motivate my students. Every day is a different attempt.* (Teacher 4B)

On the other hand, the non-material perspective surfaced in the invisible part of the relationship between work and personal life, in emotional terms, being the most dramatic and harmful of the two dimensions identified. Therefore, the issue is no longer talking about taking work home in a material sense but taking “the” work home in an emotional sense, suffering due to it^17^. Of course, for some teachers, the very act of taking work home (material dimension) is already a source of suffering, reminding us that, in the dynamics of life as it is, the two dimensions of the problem are interconnected.

Studies dedicated to analyze the issue of the mental health of teachers stress the high emotional burden and psychic suffering that these professionals face^20^^,^^21^^,^^22^. The best known may be those that address the theme from the perspective of burnout syndrome^11^^,^^23^. This is a very widespread notion; however, we prefer to observe the problem according to the concept of mental weariness^20^, a more complex and complete concept. The non-material (emotional) invasion of life by work – with the characteristically pathogenic suffering^17^ it has shown to cause – was manifested in four specific forms described below.

### Continuous Link to Work due to Successive Frustrations

Data analysis showed that teachers are subjected daily to several physical and mental aggressions during their work. Among them, our attention was drawn to the fact that, more than low wages, long working hours and excessively full classrooms^2^^,^^5^^,^^18^^,^^19^, teachers expressed frustration with being unable to perform their work, that is, they are unable to teach. This does not concern the pedagogical results themselves but working conditions.

In their statements, the professionals mentioned having the feeling that their classes are rejected by the students. Moreover, they seem to perceive the situation as a rejection of themselves, that is, of their professional identity. Clot^24^ discusses obstructed work as an element of occupational illness due to limiting the power of action, something fundamental to the balance of health. In this case, more than having the work obstructed, it was about rejected work, something that certainly causes obstruction and compromises power of action, but goes further by inserting suffering^17^ on an ontological scale.

The literature on education has discussed school failure for a long time^25^^,^^26^. The debates were on the failure of students who attended school but could not learn. Times have changed and it seems that we are now discussing another type of failure, the failure of the teacher who goes to school but is unable to even have the students' attention to teach.

*I'm going to tell you what I feel: sometimes I'm teaching, trying to explain something and the students aren't looking at me, they're not paying attention, and then I tell myself: “My God, what am I doing? To whom am I endlessly speaking here?”. They don't want to know, and then it feels bad, you know? You think it is a waste of time […because they don't want to learn. This happens sometimes and I think that's what hurts the teacher the most, what actually makes them get ill. I do believe that it is the frustration from the classroom.* (Teacher 15A)

Many teachers emphasized that the stress and frustration of being unable to do their job is a constant and systematic occurrence. For example, when talking about how she perceived work harming her health due to the stress generated by not being able to “teach”, teacher 8A stated that:

*I think that this slowly destroys us, you know. This kind of stress that we go through; it's daily, it's constant. It destroys our mental health.* (Teacher 8A)

When combined, the statements on the subject referred us to a study that showed that teachers spend more time trying to control indiscipline rather than effectively teaching^27^. We found similar data. We thus used an illustrative comparison.

In the same way a physical injury caused by repetitive movements exists – repetitive strain injury, known as RSI –, the kind of disappointment and frustration that many teachers face can produce something similar but in the psychiatric dimension. Due to being “daily and constant”, this is a repetitive frustration. Thus, we may better understand the concept of mental weariness in teachers^20^ as a “weariness caused by repeated frustrations” – a WRF so we can use a metaphor.

### Continuous Link to Work due to Moral Harassment Disturbance

In addition to living with the daily frustration of feeling rejected by the students and being unable to perform their work, the teachers also reported cases of explicit violence, that is, verbal attacks – and even physical – while teaching. Although violence at school is manifested in several ways^28^, some are more explicit than others, something that the data in our study also identified.

We thus had to consider the daily frustrations experienced by teachers – previously described – as a form of violence. That case would be a symbolic type of violence. If such violence can cause emotional damage that leads the teacher to be bitter and continually reviving the frustrations suffered, what can we say about the explicit violence? The “glaring violence”^29^?

The reports of teachers who expressed having suffered this type of violence led us to identify in it another form of invasion of life by work, much like the one we described previously but that seems to manifest itself in a more intense and painful manner. Explicit violence – that can be physical or verbal – seems to generate such a profound and prolonged suffering (pathogenic^17^) in teachers that it comes to be perceived as irremediable, scarring the individual and becoming a moral harassment:

*But I leave the school also pulling the hair out of my head from the stress they cause me. I've been called garbage, phoney, bipolar! They don't even know what bipolar is and they called me one! […] And sometimes they hurt me for months, upon months; I remember the things a seventh grader said to my face: “You can be a good teacher in other schools, but you do not belong here”. Yes, a student told me that! It was like a slap in the face. Today he cries because he didn't know me. I was able to show him that what he said is not true; he's a kid but he knows how to hurt someone, they know how to twist the knife.* (Teacher 3C)

The experience described was shown to have such a continuous link to the teacher with the emotions triggered, that not even time seemed to mitigate the suffering. The occurrence caused him “months” of bitterness, recounting the exact words uttered against him; thus, expressing not only the emotional pain of the occasion but the prolongation of the suffering experience^17^, something also expressed in several other cases.

The devaluation of teachers by the students – that is, the moral harassment– seems to have its roots in what Kleinman et al.^30^ call social suffering, a suffering that is produced within the institutions that regulate social life, resulting from the violence produced by the very social structure.

### Continuous Link to Work due to Uninterrupted Pending Matters

Another form of the invasion of life by work was what teachers described as the experience of failing to disconnect from concerns about pending matters at work, issues to be resolved not at the work environment but in their own homes and/or other private spaces.

The difficulty here is to disengage from the feeling of duties to be fulfilled (not the content itself – which is material – from work), something that stands out as the main characteristic since it extends over periods when the individual should be connected to another type of experience. However, the individual cannot do so because forgetting the pending duties of work is impossible. The individual eventually becomes covered by a state of constant unavailability for others and for the self, leading to a conflict of contexts that yet again refers us to the pathogenic suffering discussed by Dejours^17^.

We can argue that we are addressing a situation of embarrassment in which the duties of work urge the person to remain connected to it, even when outside the work environment. This is perhaps the reason why one of the teachers stated that “*teachers never leave school*” (Teacher 5A), or that, according to another teacher, her work “*begins, but has no end*” (Teacher 4D).

When reporting how he felt about such form of invasion, one of the interviewees described the feeling:

*This time that I have to spend doing things sometimes, preparing tests – it may be a short time but weekends should be used to relax – and then there's always this concern: “I need to go home, I have to do things, I have to correct tests.” So, it influences to this extent and this part of the stress is something that we end up taking home, influencing harmony.* (Teacher 15A)

Similar situations were described by several teachers, and the main points highlighted were the problems caused by the state of unavailability (for themselves and for others) that they cause, characterizing obstruction of the enjoyment of one's very life, in addition to the obstructed work discussed by Clot^24^.

### Prolonged Link with Work due to Interference on the Course of Private Life

This facet of the phenomenon occurs by the obstruction or direct interference over the course of private life, that is, over the personal life that exists – or that at least should exist – beyond work. This occurs when the individual is prevented from or suffers an interference to perform activities he/she wishes to do because of work, even when not at it. Again, it is not about obstructed work^24^, but about life being obstructed or impaired by work, thus causing suffering^17^.

When one is unable to participate in some kind of personal activity because of one's working hours it is a completely understandable case. The concept of invasion would not fit such case. However, when work prevents or hinders one from doing what he/she wants in the time of private life, we cannot deny the classification of invasion, an invasion with pathogenic repercussions^17^:

*Sometimes, for example, someone invites me to do something and I say: “I can't, I have to do this thing.” Teaching is not a job that I can just leave on Friday like a company, it's over and I'm only back on Monday, no, this is not my case.* (Teacher 5C)

In this sense, another teacher reported:

*In my opinion, teaching is no longer for me, I realized this because of the stress that it has caused to me, so I think I can no longer teach. I like it, but you know, I want to have a social life just like other people have. […] Sometimes, for example, I have to give up on some things because I have to do school activity, even during weekends.* (Teacher 4C)

Going further on this issue, the obstruction of performing desired activities because of work also presented another aspect:

*Yes, practicing sports is something I need, I feel the desire, I dedicate attention, but unfortunately I can't do it […] because it may not look like it but the preparation of classes, the study, the correction of the material and following the students… Here at the school I teach for seven different sixth grade classes, this absorbs me.* (Teacher 9A)

Like this teacher, many interviewees mentioned the desire to practice specific activities (physical activities in this case) but could not do so because of the work. This is not necessarily due to the accumulation of positions or the lack of time (considering that this teacher did not accumulate positions), but due to a specific type of fatigue that, due to being proven as very specific, we called it “school fatigue”. This is a type of fatigue that, as the sum of the various misfortunes that permeate the work activity of teachers, exhausts them, “absorbs” and subtracts their “energy”, perhaps indicating the threshold of weariness^20^ or, as some prefer to say: burnout^11^^,^^18^^,^^23^.

Interfering with family and social relationships, many teachers narrated that such weariness often leads them to seek refuge in a state of seclusion in which they stay inside their bedroom “in silence” (Teacher 3B), or ask their family to leave them “alone”:

*This is something that causes fights between me and my wife, she says: “You have to pay more attention to the girls” and I say: “Look, today I'm very tired, it wasn't a good day at school, leave me alone here” [laughs.]. […] And it goes like this: “Today I don't have the energy,” working with teenagers is too tiring sometimes.* (Teacher 1D)

Finally, the dynamic and multiple character of these various forms of invasion of life by work must be highlighted, which showed the tendency to simultaneously project over life due to being multiple forms of the same phenomenon, appearing among regular and full day teachers. Despite the differences that exist between men and women in coping and reconciling professional and domestic demands^2^^-^^6^^,^^19^^,^^20^^,^^27^, the psychic suffering resulting from bringing *work* home affected teachers of both genders.

## CONCLUSION

The results of this study showed that the investigated problem has caused suffering and impaired the health of teachers. Since they can no longer identify returns and/or perform their work, finding meaning in their profession becomes increasingly difficult; thus, impairing the pleasure that would allow them to creatively transform such suffering. In addition, external occurrences due to a growing loss of prestige of the profession in the social environment are causing a typical picture of social suffering. References to elements of pleasure in the work, for example, were almost inexistent.

Given the suffering of social scale and pathogenic type caused by the multiform invasion of life by work, such phenomenon can be considered as another element that can help to explain the recurring clinical pictures of illnesses of teachers.
