# Supplementary material for: Multiform invasion of life by work among basic education teachers and repercussions on health
Source: Rev Saude Publica. 2019 Dec 23;54:03. doi: 10.11606/s1518-8787.2020054001547 (PMC9586446; doi:10.11606/s1518-8787.2020054001547)
Supplement: Translation

# Translation: Invasão multiforme da vida pelo trabalho entre professores de educação básica e repercussões sobre a saúde

**Authors:** Jefferson Peixoto da Silva, Frida Marina Fischer

**Affiliations:** I Fundação Jorge Duprat Figueiredo de Segurança e Medicina do Trabalho – Fundacentro. São Paulo, SP, Brasil; II Universidade de São Paulo. Faculdade de Saúde Pública. Departamento de Saúde Ambiental. São Paulo, SP, Brasil

**Keywords:** Professores Escolares, Nível de Saúde, Satisfação no Emprego, Pesquisa Qualitativa, Saúde do Trabalhador

## Abstract

**INTRODUÇÃO:**

Diversos estudos têm apontado para um cenário de precarização e adoecimento entre os professores. Entretanto, o modo como o trabalho repercute sobre a vida pessoal de professores não tem recebido significativa atenção, mesmo que lhes seja comum levar trabalho para casa.

**OBJETIVO:**

Este estudo investigou a repercussão do trabalho sobre a vida pessoal cotidiana de professores e sua implicação sobre o processo saúde-doença.

**MÉTODOS:**

Estudo qualitativo que se utilizou de entrevistas individuais semiestruturadas, complementadas por formulário de caracterização sociodemográfica. Os dados foram analisados por meio de codificação temática com auxílio do *software* MAXQDA 12. Participaram do estudo 29 professores de quatro escolas públicas das redes municipal e estadual dos ensinos regular e integral de São Paulo, além dos seus respectivos diretores.

**RESULTADOS:**

Os resultados indicaram que os agravos advindos do trabalho têm se projetado sobre a vida pessoal dos professores. Identificamos quatro formas principais de manifestação desse tipo de invasão: vinculação contínua com o trabalho por: frustrações sucessivas; abalo moral; pendências ininterruptas; e interferência sobre o curso privado da vida.

**CONCLUSÃO:**

O sofrimento de amplitude social e de tipo patogênico que a invasão da vida pelo trabalho produz apontou para este fenômeno como um dos elementos que podem ajudar a explicar os recorrentes quadros de adoecimento dos professores.

## INTRODUÇÃO

Estudos dedicados a analisar a relação trabalho-saúde de professores têm demonstrado que a organização e as condições de trabalho desses profissionais são hostis, levando-os ao adoecimento^1^^-^^6^ e/ou ao abandono da profissão em suas variadas formas^7^^,^^8^. Entre as principais formas de adoecimento apontadas por tais estudos, ganham destaque os transtornos mentais e comportamentais; os distúrbios da voz; os distúrbios musculoesqueléticos; e os problemas cardiológicos. Embora não sejam os únicos, esses desfechos configuram um quadro de doenças típicas dos professores, conforme observado também entre professores de outros países^9^^-^^11^.

Sabe-se que o trabalho do professor possui uma peculiaridade pouco visível composta pelas atividades realizadas fora da escola e do tempo regular de trabalho, como é caso da preparação de aulas, da correção de provas etc. De modo geral, o mundo do trabalho tem passado por mudanças significativas nos últimos tempos. Demandas laborais avançam cada vez mais sobre a vida privada e sobre o tempo livre das pessoas, causando impactos negativos à saúde^12^. Ainda assim, a literatura dedicada à saúde ocupacional dos docentes pouco fala sobre o assunto, concentrando seu olhar nas atividades realizadas na escola, local formal de trabalho.

Em nossa revisão, percebemos que despontaram pesquisas sobre qualidade de vida de professores na última década^13^. Nelas, contudo, o problema em questão também não é explorado. A literatura educacional, por sua vez, focada na relação ensino-aprendizagem (mediada, sobretudo, pelo estudo da formação de professores e das políticas educacionais), parece não ter incorporado a relação saúde-trabalho em sua pauta, quer dentro ou fora da escola.

Como então os professores estariam vivenciando e interpretando tal realidade? Que sentidos estariam atribuindo a essas experiências? De que forma (ou formas) o trabalho estaria se projetando sobre sua vida pessoal cotidiana e até que ponto isso estaria repercutindo nos quadros de adoecimento desses profissionais? A questão principal na qual ancoramos nossa investigação foi, portanto, a hipótese de que o trabalho dos professores estaria a invadir sua vida pessoal cotidiana de modo a prejudicar sua saúde.

## MÉTODOS

Pesquisa qualitativa de caráter exploratório realizada, empiricamente, por meio de entrevistas individuais semiestruturadas. De modo complementar, aplicou-se formulário para caracterização sociodemográfica dos participantes.

A amostra foi constituída por 29 professores de educação básica atuantes em quatro escolas públicas de São Paulo e pelos quatro diretores dessas escolas, todos em exercício. Duas escolas se dedicavam ao ensino regular (uma municipal e outra estadual), enquanto as outras duas, ao ensino integral (ambas estaduais). Os dados foram analisados por meio de codificação temática^14^^,^^15^ com auxílio do *software* MAXQDA, versão 12^16^. Na perspectiva do campo teórico da saúde do trabalhador, o estudo fundamentou-se principalmente na psicodinâmica do trabalho^17^.

Para esta concepção, a chave para a compreensão do processo saúde-doença relacionada ao trabalho reside no limiar entre dois tipos de sofrimento que o perpassam: o criativo e o patogênico. Por ser inerente à experiência humana, o sofrimento (assim como o prazer) é ubíquo, mas se ele levará ao adoecimento ou não dependerá, sobretudo, dos meios pelos quais o trabalhador conseguirá (ou não) transformar criativamente esse sofrimento. Se não o puder fazer, tal sofrimento tende a se constituir em patogênico.

O projeto foi aprovado pelo Comitê de Ética em Pesquisa da Faculdade de Saúde Pública da Universidade de São Paulo, sob o n° 1.553.835, CAAE 54839516.1.0000.5421.

## RESULTADOS E DISCUSSÃO

A média de idade dos participantes foi de 45 anos, variando entre 29 e 61 anos, e o tempo de experiência na docência entre 1 a 37 anos (média de 18 anos), constituindo público heterogêneo, conforme padrão de viabilidade delimitado. O sexo feminino compôs 55% do público.

Com relação ao tipo de vínculo, 86% eram titulares de cargo efetivo, 10% estáveis e 4% tinham contrato temporário. Referente ao estado civil, 55% eram casados e 35% solteiros, enquanto 3% eram divorciados e 7% viviam em união estável.

As jornadas de trabalho variaram de 11 a 69 horas-aulas semanais (45,6 horas-aula em média). Daqueles, cujo acúmulo de vínculos era permitido (professores do ensino integral não podem ter outra atividade remunerada no horário de funcionamento da escola), 55% acumulavam e 45% não. Dados sociodemográficos semelhantes foram observados em outros estudos^2^^,^^10^^,^^18^^,^^19^.

Quanto ao elemento central da nossa investigação (a relação entre o trabalho, a vida pessoal e a saúde), 76% dos professores informaram no formulário de caracterização dos participantes que levavam trabalho para casa. Durante as entrevistas, todavia, este número mostrou-se bem maior, já que praticamente todos os entrevistados admitiram fazê-lo.

Conforme os depoimentos demonstraram, há uma dinâmica nociva do trabalho do professor que se manifesta a partir do avanço desse trabalho sobre sua vida pessoal cotidiana, o que acontece sem que haja consentimento, escolha ou controle por parte do profissional (daí o uso do termo invasão), causando sofrimento^17^. Diversos entrevistados se referiram a tal fenômeno a partir da menção a uma grande dificuldade de conseguir se desligar da escola e dos agravos nela vivenciados, como a seguinte professora:

*Eu levo tudo para minha casa. […] O tudo que eu falo é o nervosismo que eu passo, o estresse que eu passo na sala de aula. Aí o meu marido fala assim: “PROF 11A, o portão da escola já fechou, então são outros assuntos”, mas eu não consigo me desligar, deveria ter uma tomadinha que a gente desligasse, desligou e acabou, mas comigo não acontece [risos]. Eu levo para a casa, de vez em quando eu falo para o meu marido, isso é ruim porque meu marido fala: “P*ô*, eu já tenho meu trabalho, ainda vou ter que pegar os problemas do seu trabalho também, da escola? Deixa ele lá.” E* não é isso que acontece. (PROF 11A)

A invasão da vida pelo trabalho que identificamos em nosso estudo, contudo, não ocorre de modo único e linear, mas complexo e multiforme. Basicamente, duas dimensões dessa invasão foram encontradas, uma material e outra não material.

Na perspectiva material, o fenômeno assumiu sua forma mais conhecida, pelo menos no caso dos professores. Isso porque se tratou da forma mais perceptível do problema, fato considerado comum na profissão, que é levar trabalho para casa. Chamamos aqui de invasão material por dizer respeito ao conteúdo propriamente material do trabalho, como elaboração e correção de provas, trabalhos etc.:

*Essa é a diferença, porque antes de entrar na educação eu fui bancário, chegava na sexta-feira eu passava a régua: “O fim de semana é meu, a noite é minha”. Na educação não dá pra fazer isso, você deixa parte do material aqui, mas tem muita atividade em casa para fazer. Você tem que elaborar uma prova, elaborar um trabalho, na medida do possível, e trazer no dia seguinte e pensar numa alternativa, numa forma de motivá-los, e a cada dia é uma tentativa diferente.* (PROF 4B)

Por sua vez, a perspectiva não material se revelou no invisível da relação entre o trabalho e a vida pessoal, em termos emocionais, sendo a mais dramática e nociva das duas dimensões identificadas. Não se trata mais, portanto, de falarmos sobre levar trabalho para casa, em sentido material, mas sim sobre levar “o” trabalho para casa, em sentido emocional, sofrendo com isso^17^. Claro que, para alguns professores, o próprio ato de levar trabalho para casa (dimensão material) já se constitui fonte de sofrimento, nos ajudando a lembrar que, na dinâmica da vida como ela é, as duas referidas dimensões do problema encontram-se interligadas.

Estudos dedicados a analisar a questão da saúde mental do professor colocam em evidência a alta carga emocional e o sofrimento psíquico que esses profissionais têm enfrentado^20^^,^^21^^,^^22^. Os mais conhecidos talvez sejam aqueles que abordam o assunto a partir da perspectiva da síndrome de *burnout*^11^^,^^23^. Trata-se de noção bastante disseminada, ainda que prefiramos olhar para o problema segundo o conceito de desgaste mental^20^, que é mais complexo e completo. A invasão não material (emocional) da vida pelo trabalho, com o sofrimento caracteristicamente patogênico^17^ que ela demonstrou gerar, manifestou-se sob quatro formas específicas, descritas a seguir.

### Vinculação Contínua com o Trabalho por Frustrações Sucessivas

A análise dos dados revelou que os professores estão sendo submetidos diariamente a uma série de agressões físicas e mentais durante a realização do seu trabalho. Entre elas, chamou-nos a atenção o fato de que, mais do que baixos salários, longas jornadas e salas cheias^2^^,^^5^^,^^18^^,^^19^, os docentes se mostraram frustrados com o fato de não estarem conseguindo realizar seu trabalho, isto é, de não estarem conseguindo lecionar. Isso não diz respeito aos resultados pedagógicos propriamente ditos, mas às condições de trabalho.

Em seus depoimentos, os profissionais mencionaram ter a sensação de que suas aulas vinham sendo rejeitadas pelos alunos. Para além desse fato, eles parecem estar percebendo a situação como uma rejeição a si próprios, isto é, à sua identidade profissional. Clot^24^ fala do trabalho impedido como elemento de adoecimento ocupacional, por tolher o poder de agir, que é fundamental para o equilíbrio da saúde. Neste caso, mais do que ter o trabalho impedido, tratou-se de trabalho rejeitado, algo que, decerto, produz impedimento do trabalho e compromete o poder de agir, mas vai além ao inserir o sofrimento^17^ em escala ontológica.

Na literatura educacional, durante muito tempo se falou sobre o fracasso escolar^25^^,^^26^. Na ocasião, se debatia o fracasso do aluno que ia à escola, mas não conseguia aprender. Os tempos mudaram e parece que agora estamos a falar de outro tipo de fracasso, que é o fracasso do professor, que vai à escola, mas não consegue nem ter a atenção dos alunos para ensinar.

*Vou falar o que eu sinto: às vezes estou dando aula, estou tentando explicar e o pessoal não está olhando,* não está prestando atenção, e aí eu falo: “*Meu Deus, o que eu estou fazendo? Pra quem eu estou aqui falando e falando?”. Eles não querem saber, e daí dá uma sensação ruim, sabe? Você acha um tempo perdido […] porque eles não estão querendo aprender. Isso às vezes acontece e acho que isso é o que mais machuca o professor e faz com que ele adoeça mesmo. Eu penso assim que é a frustração da sala de aula.* (PROF 15A)

Muitos professores frisaram que o estresse e a frustração de não estarem conseguindo realizar seu trabalho é algo constante e sistemático. Por exemplo, ao falar sobre como percebia o trabalho prejudicando sua saúde em função do estresse gerado por não conseguir “dar aula”, a professora 8A afirmou o seguinte:

*Eu acho que isso vai acabando com a gente, entendeu? Esse tipo de estresse que a gente passa, esse tipo de nervoso, que é diário e constante, acaba com a saúde psicológica da gente.* (PROF 8A)

Combinados, os relatos referentes ao assunto nos remeteram a um estudo que demonstrou que os professores ficavam mais tempo tentando controlar a indisciplina do que efetivamente lecionando^27^. Encontramos dados semelhantes. Com base nisso, lançamo-nos a uma comparação de cunho ilustrativo.

Assim como existe um tipo de lesão física que é ocasionada por movimentos e esforços repetitivos (lesão por esforços repetitivos, mais conhecida pela sigla LER), o tipo de decepção e de frustração que muitos professores estão vivenciando pode produzir algo semelhante, mas na dimensão psíquica. Por ser “diária e constante” é, portanto, uma frustração repetitiva. Assim, talvez, possamos entender melhor o conceito de desgaste mental dos professores^20^ como um “desgaste provocado por frustrações sucessivas”, um DFS, para nos servirmos aqui de uma metáfora.

### Vinculação Contínua com o Trabalho por Abalo Moral

Além de conviver cotidianamente com a frustração de se sentirem rejeitados pelos alunos e de não conseguirem realizar seu trabalho, os professores relataram também casos de violência explícita, isto é, de agressão verbal – e até física – durante o exercício da profissão. A violência na escola, embora se manifeste de formas diversas^28^, tem algumas facetas mais explícitas do que outras, fato que os dados encontrados em nosso estudo também permitiram identificar.

Desse modo, não pudemos deixar de considerar também como violência as frustrações sucessivas vividas cotidianamente pelos professores, conforme descrito no item anterior. Naquele caso, ela tenderia um pouco mais para a dimensão simbólica. Se tal tipo de violência é capaz de causar prejuízos emocionais que levam o professor a ficar amargando e revivendo continuamente as frustrações sofridas, o que diremos então daquele tipo de violência que é explícita, ou seja, que “salta aos olhos”^29^?

Os relatos de professores que manifestaram ter sofrido esse tipo de violência nos levaram a identificar nela outra forma de invasão da vida pelo trabalho, muito parecida com aquela que descrevemos anteriormente, mas que parece se manifestar de modo mais intenso e doloroso. Podendo ser física ou verbal, essa violência explícita parece gerar nos professores um sofrimento de tipo tão profundo e prolongado (patogênico^17^) que chega a parecer irremediável, deixando marcas e assumindo a forma de um abalo moral:

*Mas eu saio daqui também arrancando cabelo da minha cabeça do estresse que eles me deixam. Eu já fui chamado de lixo, de falso, de bipolar! Eles nem sabem o que é bipolar, e eles falaram! […] E* às vezes eles me machucam de meses, meses, de eu lembrar da palavra que um aluno de sétimo ano disse na minha cara: “*Você pode ser bom professor nas outras escolas, aqui você não serve”.* É*, aluno falou pra mim! Isso foi como se fosse um tapa na minha cara. Hoje ele chora, porque não me conhecia. Eu consegui mostrar pra ele que o que ele falou não é verdade, então é criança, mas ele sabe magoar, eles sabem enfiar a faca.* (PROF 3C)

A experiência descrita demonstrou ter produzido uma vinculação tão contínua do professor com as emoções desencadeadas que nem mesmo o tempo parecia estar conseguindo atenuar. Foi algo que o levou a ficar “meses” remoendo as exatas palavras proferidas contra ele, expressando não apenas a dor emocional da ocasião, mas o prolongamento da experiência de sofrimento^17^, tal qual expresso em diversos outros casos.

Refletindo desvalorização dos alunos para com seus professores, o abalo moral em questão parece ter suas raízes naquilo que Kleinman et al.^30^ chamam de sofrimento social, um sofrimento que é produzido no seio das instituições que regulam a vida em sociedade, resultando da violência produzida pela própria estrutura social.

### Vinculação Contínua com o Trabalho por Pendências Ininterruptas

Outra forma de invasão da vida pelo trabalho identificada foi aquilo que os professores descreveram como a experiência de não conseguirem se desligar da preocupação com pendências do trabalho, questões de trabalho a serem resolvidas não no trabalho, mas nas suas próprias casas e/ou outros espaços privados.

Aqui, é a dificuldade em se desvencilhar da sensação de deveres a cumprir (e não o conteúdo em si – que é material – do trabalho) que se destaca como característica principal, uma vez que ela se estende sobre momentos em que a pessoa deveria estar conectada a outro tipo de experiência. Entretanto, ela não consegue fazê-lo, porque se esquecer dos deveres pendentes do trabalho não é possível. Assim, ela acaba envolvida por um estado de indisponibilidade constante para os outros e também para si, levando a um conflito de contextos, o que nos remete novamente ao sofrimento patogênico de que fala Dejours^17^.

Podemos dizer que estamos abordando uma situação de constrangimento na qual os deveres do trabalho instam a pessoa a continuar ligado a ele, mesmo quando fora do ambiente. Talvez seja por isso que um dos professores afirmou que “*o professor nunca sai da escola*” (PROF 5A) ou que, segundo outra professora, seu trabalho “*tem começo, mas não tem fim*” (PROF 4D).

Ao se referir ao modo como vivenciava tal forma de invasão, um dos entrevistados narrou a sensação:

*Esse tempo que eu tenho que passar às vezes fazendo as coisas, produzindo, preparando provas – embora seja pouco tempo, mas final de semana seria para relaxar – e aí tem sempre essa preocupação: “Preciso voltar pra casa, tenho o que fazer, tenho que corrigir prova”. Então influencia nesse ponto e essa parte do estresse que a gente acaba levando pra casa e influencia na harmonia.* (PROF 15A)

Situações semelhantes foram descritas por diversos professores, e os principais pontos ressaltados foram os problemas causados pelo estado de indisponibilidade (para si e para o outro) que elas geram, caracterizando, para além do trabalho impedido de que fala Clot^24^, o impedimento da fruição da própria vida.

### Vinculação Prolongada com o Trabalho por Interferência Sobre o Curso da Vida Privada

Tal faceta do fenômeno se dá pelo impedimento ou pela interferência direta sobre o curso da vida privada, isto é, sobre a vida pessoal que existe – ou que pelo menos deveria existir – para além do trabalho. Acontece quando o indivíduo é impedido ou sofre interferência na realização de atividades que deseja fazer por causa do trabalho, mesmo não estando nele. Novamente, não se trata de trabalho impedido^24^, mas da vida impedida ou prejudicada pelo trabalho, gerando sofrimento^17^.

Quando não se pode fazer algum tipo de atividade pessoal devido ao horário de trabalho, o fato é plenamente compreensível. Nesse caso, não caberia falar em invasão. No entanto, quando ele impede ou atrapalha fazer o que se deseja no tempo da vida privada, não tivemos como negar a classificação como invasão, uma invasão com repercussões patogênicas^17^:

À*s vezes, por exemplo, alguém te chama para fazer alguma coisa e você fala: “N*ão posso, tenho que fazer tal coisa”*. N*ão é um trabalho que eu saio, como se fosse uma empresa, acabou, só na segunda-feira, no meu caso, não. (PROF 5C)

No mesmo sentido, outro professor relatou:

*Eu acho que assim, o magistério já não é mais pra mim, porque eu já percebi isso pelo estresse que ele tem me gerado, então eu acho que ele não dá mais. Eu gosto, mas assim, eu queria ter uma vida social igual às outras pessoas têm. […] Eu às vezes, por exemplo, tenho que me abdicar de algumas coisas porque eu tenho que fazer atividade de escola, inclusive no final de semana.* (PROF 4C)

Aprofundando a questão, o impedimento de realizar atividades desejadas por causa do trabalho apresentou também outro aspecto:

*Pois é, a questão da prática do esporte é uma questão que eu necessito, eu sinto o desejo, dedico atenção, mas infelizmente não estou podendo fazer […] porque parece que não, mas a preparação de aulas, o estudo, a correção do material e você acompanhar os alunos… Aqui na escola eu leciono para sete sextos anos, então isso me absorve.* (PROF 9A)

Assim como esse professor, muitos entrevistados mencionaram o desejo de praticar atividades específicas (como, no caso, atividades físicas), mas não conseguir fazê-lo por conta do trabalho. Isso não necessariamente pelo acúmulo de cargos ou pela falta de tempo em si (até mesmo porque o professor em questão não acumulava cargos), mas devido a um tipo específico de cansaço que, por ter se demonstrado muito específico, chamamos de “cansaço de escola”. Trata-se de um cansaço que, como soma dos diversos infortúnios que têm perpassado a atividade de trabalho dos professores, esgota o docente, o “absorve” e lhe subtrai a “disposição”, indicando talvez o limiar do desgaste^20^ ou, como alguns preferem frisar, do *burnout*^11^^,^^18^^,^^23^.

Interferindo nas relações familiares e sociais, muitos professores narraram que tal cansaço muitas vezes os leva a buscarem se refugiar em um estado de reclusão no qual se fecham no quarto e ficam “em silêncio” (PROF 3B), ou pedem à família que lhes deixem “quietinhos”:

*Isso é motivo de discussão com a minha esposa, de ela falar: “Você precisa dar mais atenção para as meninas”, e eu falar: “Olha, hoje estou muito cansado, não foi um dia bom na escola, deixa eu quietinho aqui” [risos.]. […] E aí fica essa coisa: “Hoje não estou com muita disposição”, trabalhar com adolescente às vezes é muito pesado.* (PROF 1D)

Há que se destacar, por fim, o caráter dinâmico e múltiplo dessas diversas formas de invasão da vida pelo trabalho, as quais demonstraram a tendência de se projetarem simultaneamente sobre a vida, visto se tratar de múltiplas formas de um mesmo fenômeno, revelando-se tanto entre professores do ensino regular quanto do ensino integral. A despeito das diferenças que existem entre homens e mulheres no enfrentamento e conciliação de demandas profissionais e domésticas^2^^-^^6^^,^^19^^,^^20^^,^^27^, o sofrimento psíquico decorrente de levar *o trabalho* para casa atingiu professores de ambos os gêneros.

## CONCLUSÃO

Os resultados deste estudo demonstraram que o problema investigado tem causado sofrimento e prejudicado a saúde dos professores. Por não estarem conseguindo ver retorno e/ou realizar o seu trabalho, vai ficando cada vez mais difícil encontrar sentido e, portanto, prazer que lhes permita proceder à transformação criativa desse sofrimento. Somado a isso, impulsos externos decorrentes de um crescente desprestígio da profissão no meio social vêm gerando um quadro típico de sofrimento social. Menções a elementos de prazer no trabalho, por exemplo, foram praticamente inexistentes.

Dado o sofrimento de amplitude social e de tipo patogênico que a invasão multiforme da vida pelo trabalho produz, tal fenômeno pode ser considerado como mais um dos elementos que podem ajudar a explicar os recorrentes quadros de adoecimento dos professores.
